# Post-stroke deficits in the anticipatory control and bimanual coordination during naturalistic cooperative bimanual action

**DOI:** 10.1186/s12984-023-01257-x

**Published:** 2023-11-10

**Authors:** Cory A. Potts, Shailesh S. Kantak

**Affiliations:** 1 Moss Rehabilitation Research Institute, Elkins Park, PA USA; 2 Department of Psychology, State University of New York at Plattsburgh, Plattsburgh, USA; 3 Department of Physical Therapy, Arcadia University, Elkins Park, PA USA

**Keywords:** Bimanual, Stroke, Cooperative coordination, Motor planning

## Abstract

**Background:**

Unilateral stroke leads to asymmetric deficits in movement performance; yet its effects on naturalistic bimanual actions, a key aspect of everyday functions, are understudied. Particularly, how naturalistic bimanual actions that require the two hands to cooperatively interact with each other while manipulating a single common object are planned, executed, and coordinated after stroke is not known. In the present study, we compared the anticipatory planning, execution, and coordination of force between individuals with left and right hemisphere stroke and neurotypical controls in a naturalistic bimanual common-goal task, lifting a box.

**Method:**

Thirty-three individuals with chronic stroke (15 LCVA, 18 RCVA) and 8 neurotypical age-matched controls used both hands to lift a box fitted with force transducers under unweighted and weighted conditions. Primary dependent variables included measures of anticipation (peak grip and load force rate), execution (peak grip force, load force), and measures of within-hand (grip-load force coordination) and between-hand coordination (force rate cross-correlations). Primary analyses were performed using linear mixed effects modeling. Exploratory backward stepwise regression examined predictors of individual variability within participants with stroke.

**Results:**

Participants with stroke, particularly the RCVA group, showed impaired scaling of grip and load force rates with the addition of weight, indicating deficits in anticipatory control. While there were no group differences in peak grip force, participants with stroke showed significant impairments in peak load force and in grip-load force coordination with specific deficits in the evolution of load force prior to object lift-off. Finally, there were differences in spatial coordination of load force rates for participants with stroke, and especially the RCVA group, as compared to controls. Unimanual motor performance of the paretic arm and hemisphere of lesion (right hemisphere) were the key predictors of impairments in anticipatory planning of grip force and bimanual coordination among participants with stroke.

**Conclusions:**

These results suggest that individuals with stroke, particularly those with right hemisphere damage, have impairments in anticipatory planning and interlimb coordination of symmetric cooperative bimanual tasks.

**Supplementary Information:**

The online version contains supplementary material available at 10.1186/s12984-023-01257-x.

Activities of daily living are replete with goal-directed actions that engage both upper extremities in different spatiotemporal interrelationships to ensure efficient performance [1, 2]. Following unilateral stroke, many survivors show impaired function of the upper extremities, contributing to disability and discontinuation of daily activities [3]. While a considerable body of research has examined sensorimotor impairments and performance of the paretic arm after stroke [4], there is mounting evidence for performance deficits in the nonparetic arm [5]. Stroke also affects interhemispheric connections, and higher-level motor planning regions that influence how skilled bimanual actions are planned and executed [6]. It is therefore of clinical and scientific interest to determine if the planning and coordination of bimanual skills is affected in individuals with unilateral stroke, and to assess the degree to which sensorimotor deficits of the paretic arm influence bimanual coordination.

Previous research on bimanual coordination after stroke has mainly investigated rhythmic or discrete bimanual actions where each arm/hand acts independently to manipulate a separate object or accomplish a distinct movement subgoal (e.g., 7). Further, key studies investigating inter-limb coordination during isometric bimanual force control tasks have demonstrated poor accuracy, variability and impaired coordination between hands after stroke [7–10]. These investigations have provided crucial insights into the mechanisms of coupling and interference between the two arms, and the effect of stroke [11–14]. Compared to such experimental tasks, however, real-world bimanual actions are often more complex, requiring the two arms to collaborate and compensate for each other while also adapting to object properties [15, 16]. For example, while lifting a box with both hands, each hand must coordinate with the other to exert accurate forces dependent on object weight to grip and lift the box successfully. Such cooperative coordination between the hands to accomplish a common goal is likely supported by neural substrates that are distinct from those involved with bimanual coordination for independent goals. Previous research has demonstrated reduced intracortical inhibition and higher interhemispheric inhibition for bimanual common-goal as compared to independent-goal tasks in neurotypical adults during isometric force-production task [16, 17]. With distinct neurobehavioral mechanisms implementing distinct modes of coordination and the preponderance of cooperative bimanual actions in daily life, it is important to determine how cooperative naturalistic actions are planned and performed after unilateral stroke.

Previously we used kinematic analyses to determine how individuals with stroke coordinated their hands while reaching and picking up a box [18]. The results showed that, while reaching toward the box, participants with stroke coupled their arms similar to neurotypical controls. This, like other studies, suggested that parallel coordination to transport the hands to the box was relatively unimpaired after stroke. However, the time from box contact to box pick-up (pickup time) was significantly prolonged in the stroke group. Prolonged pickup times likely indicate impairments in cooperative coordination where the forces of each hand were planned and coordinated with the other to grip and lift the box. We did not collect kinetic data in that study, however, which precluded inferences about the anticipatory planning, execution and coordination of forces underlying deficient performance of lifting the box.

While lifting a box, the two hands must cooperatively interact to apply perpendicular grip force (GF) to secure the box between hands before applying tangential load force (LF) to counteract the forces of gravity and lift the box [15, 19, 20]. This cooperative coordination is dependent on the ability to plan necessary grip and load forces in an anticipatory manner and execute them for successful pickup. Anticipatory planning during pickup is driven by the sensorimotor memory of object properties such as object weight developed over 1–2 previous trials [20–23] and is reflected in the scaling of early force metrics such as the grip force rate (GFR) and load force rate (LFR) to object weight. GFR and LFR peak prior to object lift when more robust feedback of the object is available and therefore, are thought to reflect anticipatory planning [20]. As the object lift is executed, the GF and LF of each hand are tightly coordinated, increasing in smooth trajectories with the onset and increase of GF slightly preceding that of LF in neurotypical individuals [24]. Finally, to ensure that the box is lifted with minimal tilt, the relative positions and forces of the two hands are coordinated in time and space - a hallmark of cooperative coordination. Such cooperative coordination between the hands relies on the ability to sense and integrate haptic feedback across both hands and adjust forces accordingly to ensure successful box pick up [25]. Sensorimotor impairments such as unimanual paresis and sensory deficits after stroke may thus impair planning, execution and coordination between hands. The literature has thus far yielded conflicting evidence, with some reports suggesting a strong relationship between paretic arm motor impairment (e.g., Upper Extremity Fugl-Meyer, UEFM) and bimanual coordination [6], while others have failed to demonstrate such a relationship [18]. Determining the influence of sensorimotor impairments of the paretic arm on a cooperative bimanual task may help identify patients to target appropriate therapies to those deficits and improve bimanual task performance.

Previous research in unimanual actions has identified hemisphere-specific deficits in the paretic and nonparetic arm. Specifically, after left cerebrovascular accident (LCVA), anticipatory planning deficits in the early phase of movement are robustly evident, whereas after right cerebrovascular accident (RCVA), feedback-based online control in the later phases of movement is deficient during unimanual actions. For bimanual actions, evidence from neuroimaging as well as patient studies for specialized role for each hemisphere is conflicting. For example, in neurotypical adults, bimanual common-goal tasks, as compared to independent-goal tasks, are associated with greater activation of right-hemisphere structures including primary motor cortex (M1), supplementary motor area (SMA), and superior temporal gyrus (STG) [26]. Additionally, transient disruption to right STG selectively impaired performance for the common-goal task, but not independent-goal task, implying a causal role of right STG for bimanual common-goal tasks. In contrast, Schaffer and colleagues found that during a bilateral reaching task moving a common cursor, individuals with left hemisphere damage showed deficits in early phases of movement. This indicated the role of left hemisphere in anticipatory control of common-goal actions [27]. Thus, the extent to which the lesion of hemisphere influences planning, execution and coordination of cooperative bimanual actions is not clear and needs investigation to help identify patients and apply therapy targets in an individualized manner.

The goal of the present research was to determine how individuals with unilateral stroke affecting the left and right hemisphere plan, execute and coordinate the forces between the two arms during a cooperative bimanual task of picking up a box, compared to age-matched neurotypical controls. Individuals with LCVA, RCVA and age-matched controls lifted a box fitted with force transducers while the weight of the box was varied. Planning was assessed by measurement of anticipatory scaling of peak LFR and peak GFR to object weight, as the peak amplitude of these variables is scaled to the expected weight of the object before sensory feedback of the object’s weight is available at lift-off [22, 28, 29]. Given the specialized role of the left-hemisphere in anticipatory planning, we hypothesized that scaling of GFR and LFR to object weight will be impaired in the LCVA group compared to controls and RCVA group. Execution was assessed by measurement of peak load force, peak grip force and the coordination between grip and load force. Given the specialized role of the right hemisphere in feedback-based online execution, we hypothesized that peak load, grip forces as well as the coordination between grip and load forces will be more impaired in the RCVA group compared to the control and LCVA group. Bimanual coordination was assessed by measuring the spatial and temporal synchrony of grip and load force rate profiles between the two hands. Based on the prior findings indicating the role of the right hemisphere in cooperative bimanual coordination [26], we hypothesized that individuals with RCVA will demonstrate reduced spatial and temporal synchrony in grip and load forces between the two arms relative to controls and LCVA. Finally, we examined the extent to which measures of sensorimotor impairments (Action Research Arm Test, fine touch, proprioception) are related to anticipatory planning, execution, and bimanual coordination during cooperative task such as picking up a box. We hypothesized that sensorimotor impairments would be more strongly predictive of deficiencies in execution and bimanual coordination than anticipatory planning, as planning is less reliant on online sensory feedback related to the physical properties of the box.

## Method

### Participants

33 participants with stroke (15 LCVA *M*_*Age*_ = 61.4, 4 females; 18 RCVA, *M*_*Age*_ = 59.44, 9 females) and 8 neurotypical age-matched controls (*M*_*Age*_ = 58.6 years, 1 female) completed the study. This sample size was derived from related work in unimanual grasping from Raghavan, Krakauer, & Gordon (2016; n = 8 in each group), though we increased the sample for participants with stroke to examine hemispheric differences. Participants were recruited from the Moss Rehabilitation Research Institute participant registry and from e-mail responses to distributed flyers posted at Arcadia University. Eighty-two individuals with stroke were screened. We included participants with stroke: [1] at least 6 months post-stroke with unilateral, first ischemic, or hemorrhagic anterior circulation stroke affecting cortical, internal capsule, or corona radiata regions; [2] with scores of 24 or more on the Standardized Mini Mental State Examination (SMMSE) or, for aphasic patients, scores of 4 or more on the Western Aphasia Battery (WAB) Auditory Comprehension subtest; [3] who transferred at least 10 blocks in one minute on the Box and Block Test [30] using the affected hand; and [4] with no upper extremity pain or musculoskeletal problems. We excluded participants with stroke with [1] visual neglect as tested by the Line Bisection Test of the Comprehensive Aphasia Battery (CAT) [31]; [2] joint pain in the affected arm at rest or in motion; [3] medical, neurological, or psychiatric conditions known to impact task performance; [4] comprehension deficits impeding task performance; [5] bilateral, brainstem, or cerebellar stroke; [6] complete paralyses or hemiplegia; and [11] use of pacemakers, defibrillators, or similar medical implants that may interfere with magnetic marker system. All subjects gave their informed consent according to the Declaration of Helsinki and were naïve to the purpose of the study.

### Apparatus

A custom-made device designed to resemble an opaque box (15.2 × 15.2 × 15.2 cm) was used to measure the forces of the right and left hand. The sides of the device equipped with carbon fiber plates that were connected to two force transducers (Mini 45Ti ATI Industrial Automation FTI8553 Multi-Axial XDCR)- one on each side to measure the applied grip and load forces (3/800 lbf). The inside of box had a central and two lateral compartments to secure movable weights using Velcro. The rear corners of the box were instrumented with magnetic tracking systems (3D-guidance trakSTAR) to determine the vertical position and tilt of the box. Kinematic data were sampled at 200 Hz and filtered using a zero-phase lag, low-pass fourth-order Butterworth filter with a cut-off frequency of 10 Hz [18, 32].

### Procedures

At the start of the testing session, participants underwent clinical testing that included measures of global cognition, handedness, motor impairment, grip strength, fine touch, proprioception, and task switching. Global cognition was measured using the Standardized Mini-Mental State Examination. Handedness was measured using the Edinburgh Handedness Inventory. Motor impairment was indexed using the Action Research Arm Test (ARAT) and Upper Extremity Fugl-Meyer scale (UEFM). Participants then performed a grip strength assessment using a JAMAR Hand Grip Dynamometer for three trials on each hand. Fine-touch sensation in each hand was assessed using Semmes Weinstein monofilament testing. Proprioceptive ability was determined using an elbow position matching task with a greater discrepancy between the passively positioned paretic arm and the actively matched non-paretic arm indicating greater proprioceptive deficits. Parts A and B of the Trail-Making Test were administered to assess executive function including attentional switching, mental flexibility, and visual scanning [30]. For a summary of these measures along with group demographics, see Table 1.

**Table 1:** Group characteristics

	Left-Hemisphere Damage (n = 15)	Right-Hemisphere Damage (n = 18)	Controls (n = 8)
Sex	~ 27% female	50% female	12.5% female
Age (years)	61.4 (2.35)	59.44 (2.12)	58.62 (2.18)
Standardized Mini-Mental State Examination (/30)	27.88(0.64)	28.94(0.34)	29.75(0.16)
Edinburgh Handedness Inventory (/100)	9.33(19.89) *	84.71(6.35) *	92.5(4.11)
Trail-Making Test B minus A (seconds)	95.14 (16.11) *	47.67 (8.53) *	29.85 (4.16)
Years post-stroke	7.79 (1.20)	5.49 (0.92)	
Action Research Arm Test (/57)	46.07(3.75)	42.12(3.58)	
Upper Extremity Fugl-Meyer (/66)	52.33(3.15)	51.66 (2.56)	
Grip Strength Ratio	0.76 (0.09)	0.68 (0.09)	
Fine touch: Semmes Weinstein monofilament test (Paretic side, /6)	3.82 (0.23)	4.31 (0.27)	
Proprioceptive Difference Score (degrees)	6.00 (0.86)	6.06 (0.93)	

Data are presented as mean (standard error of the mean). There were no significant differences between the LHD and RHD groups on any measure except for the Trail-Making Test (p = .016) and Edinburgh Handedness Inventory (p = .002) *

The experiment was completed on a separate day after clinical testing. Prior to testing, we placed markers for motion tracking on the wrist of the left and right hands. Participants were seated comfortably in front of a table such that their elbows were supported, and hands with clasped fingers rested on the table at 20% of their maximal arm reach distance (paretic arm for participants with stroke). The box was placed at 50% of their maximum paretic arm reach. The task goal was to reach and lift the box while minimizing any box tilt. When the experimenter said ‘go’, participants were instructed “to reach to place your palms on the sides of the box and pick-up the box to about shoulder height as fast and as smoothly as you can without tilting the box.” The hand placement was standardized to the center of the lateral surface and visually inspected at each trial. Participants lifted the box to approximately shoulder height, held it for three seconds, and placed it back on the tabletop. The experimenter counted the three seconds aloud and visually monitored the box for appropriate location of hands, object placement, and absence of an obvious tilt.

Participants completed a total of 15 trials for this experiment. During the first five trials, the box was unweighted. For the remaining 10 trials, the box contained a 2 lb. (0.91 kg) weight placed in the center of the box. The weight was added before the 6th trial and secured to the floor of the box with a Velcro strip. Participants were not aware of the added weight. Before each trial began, a poster board occluded their view of the box. The weight in the testing apparatus and trial sequence was consistent across all participants.

### Data analyses

For each trial, force data from the transducers and position data of the box and hand were sampled at 400 Hz and 200 Hz respectively. Force data were filtered using a second-order low pass Butterworth filter with a cutoff frequency of 10 Hz. Below we define our dependent variables of interest.

**Measures of Anticipatory planning and Execution**. Anticipatory planning was characterized by the scaling of peak grip and load force rates prior to lift onset before performance-specific feedback mechanisms influenced force control. Lift onset was defined as the point at which the object was lifted above 0.25 cm and subsequently remained above this value. Peak force rate was defined as the highest point in the force rate profile that was followed by a subsequent drop of at least 50%. In secondary analyses, we examined individual variation in the scaling of grip and load force rate among participants with stroke by calculating a difference score for peak GFR and LFR values across weight conditions (weighted – unweighted) for each participant. Execution measures were identified as peak grip and load forces.

**Measures of within-hand coordination**. Within-hand coordination was defined by the correlation between grip and load force, which was quantified as the Pearson’s correlation of between the GF and LF profiles for each trial from LF onset (10% of max LF) to max LF for the left and right hand, separately.

#### Measures of bimanual coordination

Bimanual coordination was quantified using cross-correlations between the left and right-hand force rate profiles from force rate onset to offset. Spatial coordination was characterized by peak cross correlation coefficient (r); with values closer to 1 indicating greater spatial coordination. Due to the non-normal distribution of correlation coefficients, we performed a Fisher transformation to convert the coefficients to Z-scores prior to the analyses, though we report cross-correlation values for clarity of interpretation. The associated time-lag at which peak cross-correlation coefficient was obtained indicated temporal coordination between the forces of the two hands. A shorter time-lag (closer to zero) indicated stronger temporal bimanual synchrony, while a negative lag indicated that the nondominant hand lagged behind the dominant hand.

**Tilt and Hand Position**. Box tilt was defined as the difference in Z position (cm) between the left and right side of the box. Positive tilt values indicated that the left side of the box was higher than the right (rightward tilt), while negative values indicated that the right side of the box was higher than the left (leftward tilt). Hand position was defined via motion tracking from the markers attached to the wrist of the left and right hand.

### Statistical analyses

Statistical analyses were performed in R using the *lme4* package (33). Trial-wise data were analyzed using linear mixed effects modeling (LME). Significant main effects were evaluated using likelihood ratio tests comparing a model with the effect of interest against a model without the effect of interest (which approximately follows a χ^2^ distribution). For each primary dependent variable (measures of anticipatory planning, execution and within-hand coordination), we fit a model with fixed effects for Group (Control, LCVA, RCVA), Weight Condition (Unweighted, Weighted), Hand (Right, Left), terms for all possible two and three-way interactions between these factors, and a random intercept for Subject. For measures of bimanual coordination, we fit a model with fixed effects for Group, Weight Condition, the interaction between these factors, and a random intercept for Subject. For analyses involving tilt and hand position differences, we ran a one-sample t-tests to determine whether these measures differed significantly from a value of zero, indicating no tilt or no difference in Z-position between the two hands, and then examined effects of Group and Hand using LME. Post-hoc analyses were performed using the Tukey’s test to correct for multiple comparisons. In secondary analyses, we used backward stepwise regression analyses to examine the relation between sensorimotor and clinical measures and outcome measures of interest using the *olsrr* package for R. All stepwise regressions included the same predictors, including measures of sensorimotor capacity (paretic arm ARAT score, paretic arm Semmes-Weinstein monofilament test, proprioceptive difference score), hemisphere of lesion, age, and sex. We selected the paretic arm ARAT score as a measure of paretic arm motor performance rather than UEFM score due to collinearity between UEFM and monofilament scores, the primary sensory measure of interest. All dependent variables were trimmed for outliers 2.5 standard deviations above and below subject and group mean by condition and hand. The first two trials after the addition of weight are omitted from all analyses, as generally 1–2 lifts are required to scale force and force rate. Means are reported with accompanying standard deviations.

## Results

**Fig. 1:**
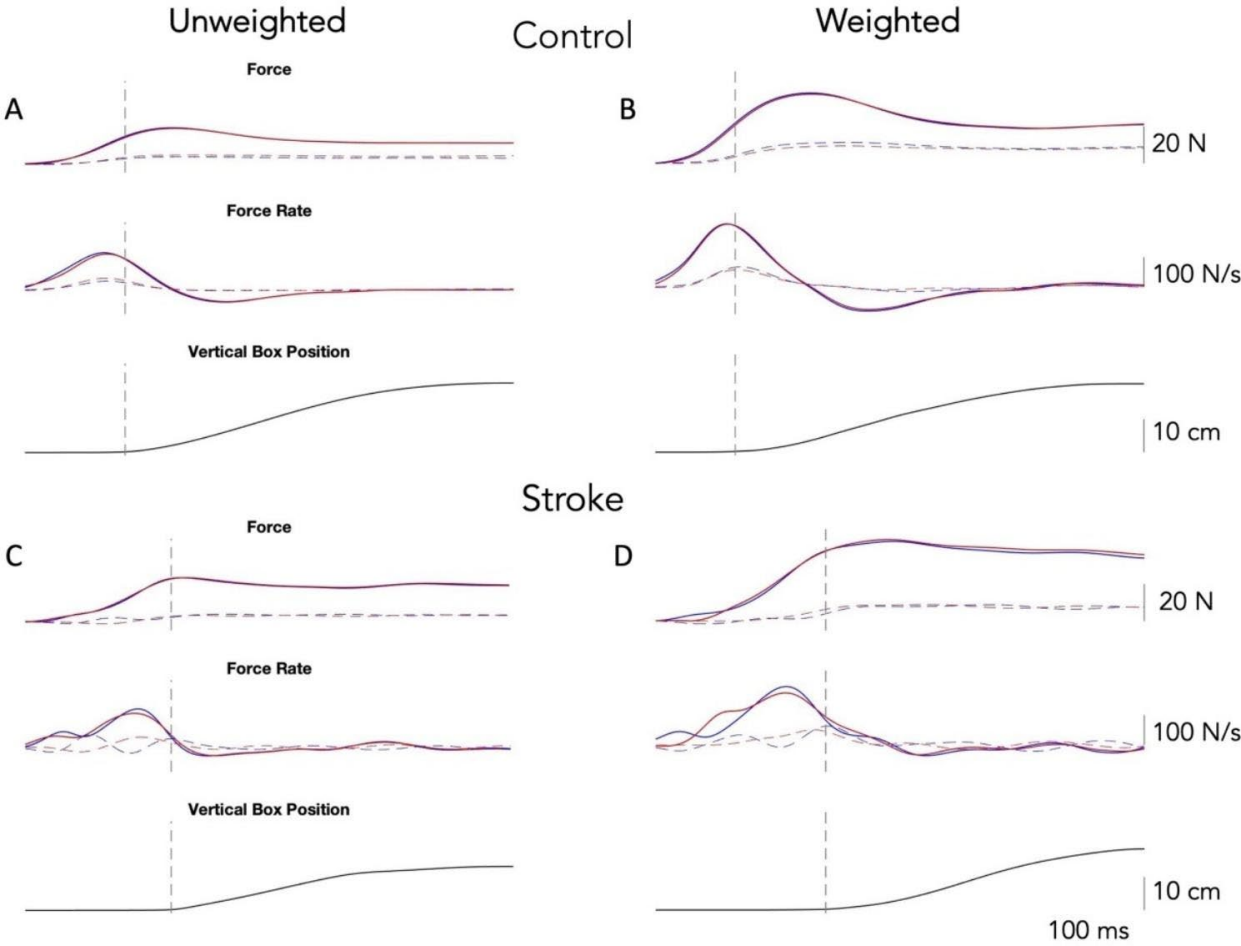
Left (blue) and right hand (red) profiles for grip force (solid) and load force (dotted), grip force rate (solid) and load force rate (dotted), and box height as a function of time for a representative neurotypical control participant and an individual with stroke (RCVA; paretic arm = left). Plots on the left show the unweighted box condition. Plots on the right show the weighted box condition. The dashed vertical line indicates the time of box lift

Figure 1 shows the profiles for grip and load force, grip and load force rate, and box height for the right (red) and left (blue) hands for a representative control participant and an individual with stroke. Left plots show unweighted box condition and right plots show the weighted condition. For the control participant, force and force rate profiles are similar between the two arms, following smooth, single-peaked trajectories. Additionally, peak force and force rates are higher for the weighted condition as compared to the unweighted box condition, with max force rates peaking before box lift. The participant with stroke (RCVA) shows deficiencies in measures of anticipation and execution for the paretic (left) arm compared to the nonparetic (right) arm, particularly in the evolution of load force and load force rate prior to box lift.

### Measures of Anticipatory Control: GFR & LFR

To confirm that force rates peaked before box lift onset, and thus can be assessed as indices of planning, we performed separate analyses testing for differences between box lift onset time and the time of peak GFR and LFR. The analyses verified that box lift onset (*M* = 2.82 s) occurred significantly later in time than peak GFR (*M* = 2.55 s), *χ*^*2*^(1) = 33.57, *p* < .001, and peak LFR (*M* = 2.63 s), χ^2^(1) = 16.98, *p* < .001.

The analysis for peak GFR and LFR tested the hypothesis that participants with LCVA would show deficient scaling of force rate across box weight conditions as compared to controls and RCVA. We also predicted that differences would emerge between the paretic arm in the stroke groups (right arm for LCVA, left for RCVA) and the nonparetic arm. To examine these effects, we analyzed LFR and GFR with linear mixed effects models including terms for Group, Weight Condition, Hand, and their interaction, as well as a random effect term for Subject.

**Fig. 2:**
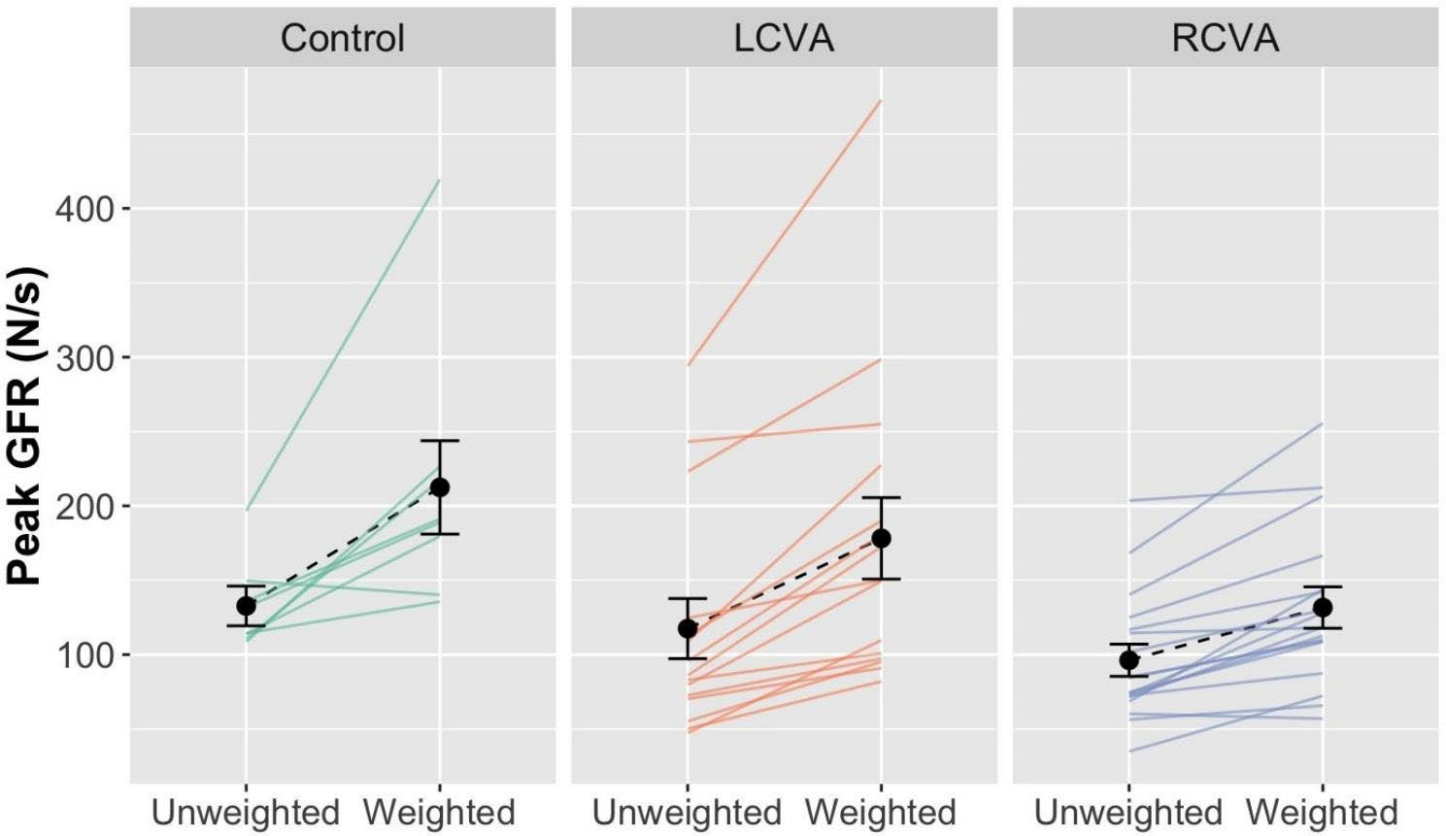
Peak GFR as a function of weight condition for the control, LCVA, and RCVA group. Solid lines show individual subject means. Dotted lines show group means. Error bars show the standard error of the mean

**Peak GFR**. The analysis of peak GFR revealed a significant Group by Weight Condition interaction, *χ*^*2*^(2) = 53.67, *p < *.001. Controls showed the greatest increase in GFR across weight conditions, followed by the LCVA group, and the RCVA group showed the smallest difference between conditions (Fig. 2). Tukey’s adjusted post-hoc contrasts examining group differences within the unweighted and weighted box conditions revealed differences between the control and RCVA groups for the weighted box condition, *p* = .02, with no other significant between-group differences. There were no additional significant two- or three-way interactions in the analysis, and thus no effects involving Hand (all p’s > 0.05). The analysis also yielded a significant main effect of Condition, *χ*^*2*^(1) = 17.54, *p < *.001, and no main effect of Group, *χ*^*2*^(1) = 5.56, *p = *.06.

As shown in Fig. 2, participants with stroke showed considerable variability in the scaling of peak GFR across weight conditions. In secondary analyses, we examined predictors of individual variation in the scaling of peak GFR using backward stepwise regression. The best fitting model, *R*^*2*^ *=* 0.30, *F* [3, 21] *=* 4.54, *p =* .01, included participants’ hemisphere of lesion (*ß = − 0.35*, *p* = .054), paretic arm ARAT score (*ß =0.32*, *p* = .09), and paretic side monofilament test score (*ß =-0.26*, *p* = .15). Importantly, the LCVA and RCVA groups did not differ across any predictors of motor or sensory function (all p’s > 0.05). The inclusion of hemisphere accords with the results for the analysis of GFR suggesting that the RCVA group was more impaired, relative to controls, than the LCVA group, for the scaling of GFR.

**Fig. 3:**
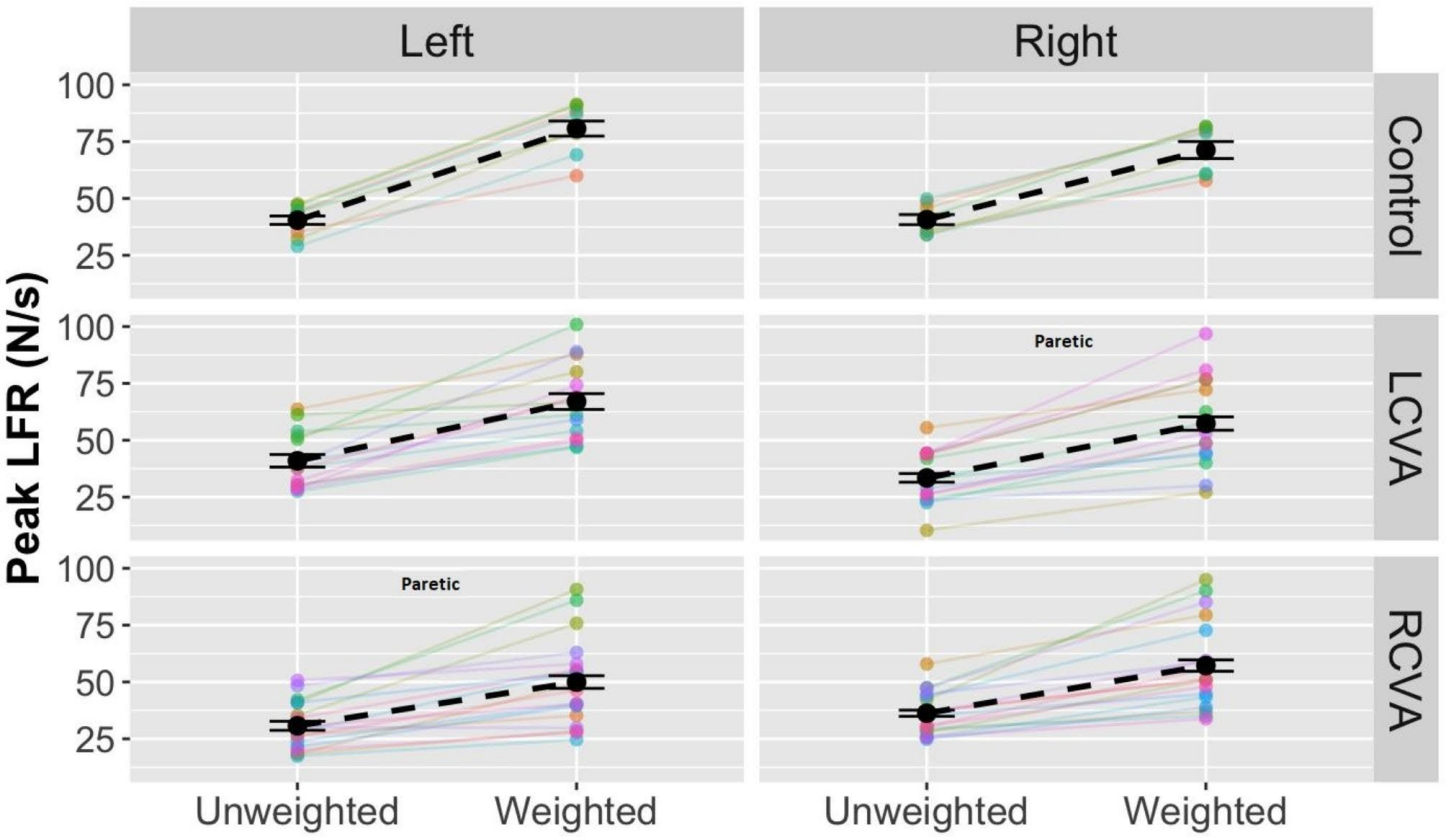
Peak LFR for the control, LCVA, and RCVA groups by Hand and Weight Condition. As indicated the right hand is the paretic hand for the LCVA group, while the left hand is the paretic hand for the RCVA group. Black points and dashed lines show group means with bars for standard error. Colored lines and points show individual participant means, with individual participants represented by a different color

**Peak LFR**. The analysis of peak LFR revealed a significant Group by Weight Condition interaction, *χ*^*2*^(2) = 38.45, *p* < .001. As shown in Fig. 3, LFR increased more steeply across weight conditions for controls as compared to the LCVA and RCVA groups. Tukey’s adjusted post-hoc contrasts examining group differences within each box weight condition revealed differences between controls and the LCVA group (*p =* .03) and the RCVA group (*p < *.001) within the weighted box condition and no other group differences. There was also a significant Hand by Group interaction, *χ*^*2*^(2) = 71.87, *p* < .001. For controls, LFR values were higher for the left hand than the right hand, while the LCVA and RCVA groups produced higher LFR with the nonparetic (left in LCVA, right in RCVA) as compared to the paretic hand (right in LCVA, right in LCVA). The interaction between Weight Condition and Hand was not significant, *p = *.38, nor was the three-way interaction between Group, Weight Condition, and Hand, *p =* .14. There were significant main effects of Hand, *χ*^*2*^(1) = 7.73, *p* < .01, Weight Condition, *χ*^*2*^(1) = 29.99, *p* < .001, and Group, *χ*^*2*^(1) = 14.72, *p* < .001.

In secondary analyses we examined individual variation among participants with stroke in the scaling of peak LFR across weight conditions (weighted – unweighted) in a stepwise regression. The final regression model was not significant (*R*^*2*^ = 0.19, *p* = .19).

### Measures of execution: GF and LF

The analysis of measures of execution, peak GF and LF, tested the hypothesis that individuals with RCVA would be impaired in the scaling of force across weight conditions as compared to controls and LCVA, and that these deficits may be more pronounced for the paretic arm in participants with stroke. To assess these effects, we fitted LME models with terms for Group, Weight Condition, Hand, the interactions between these factors, and a random effect for Subject.

**Peak GF**. A main effect of Weight Condition emerged, *χ*^*2*^(1) = 42.4, *p* < .001, with no other main effects or interactions. Peak GF was higher for the weighted (36.29 ± 9.76 N) compared to the unweighted (23.13 ± 8.22 N) box condition.

**Fig. 4:**
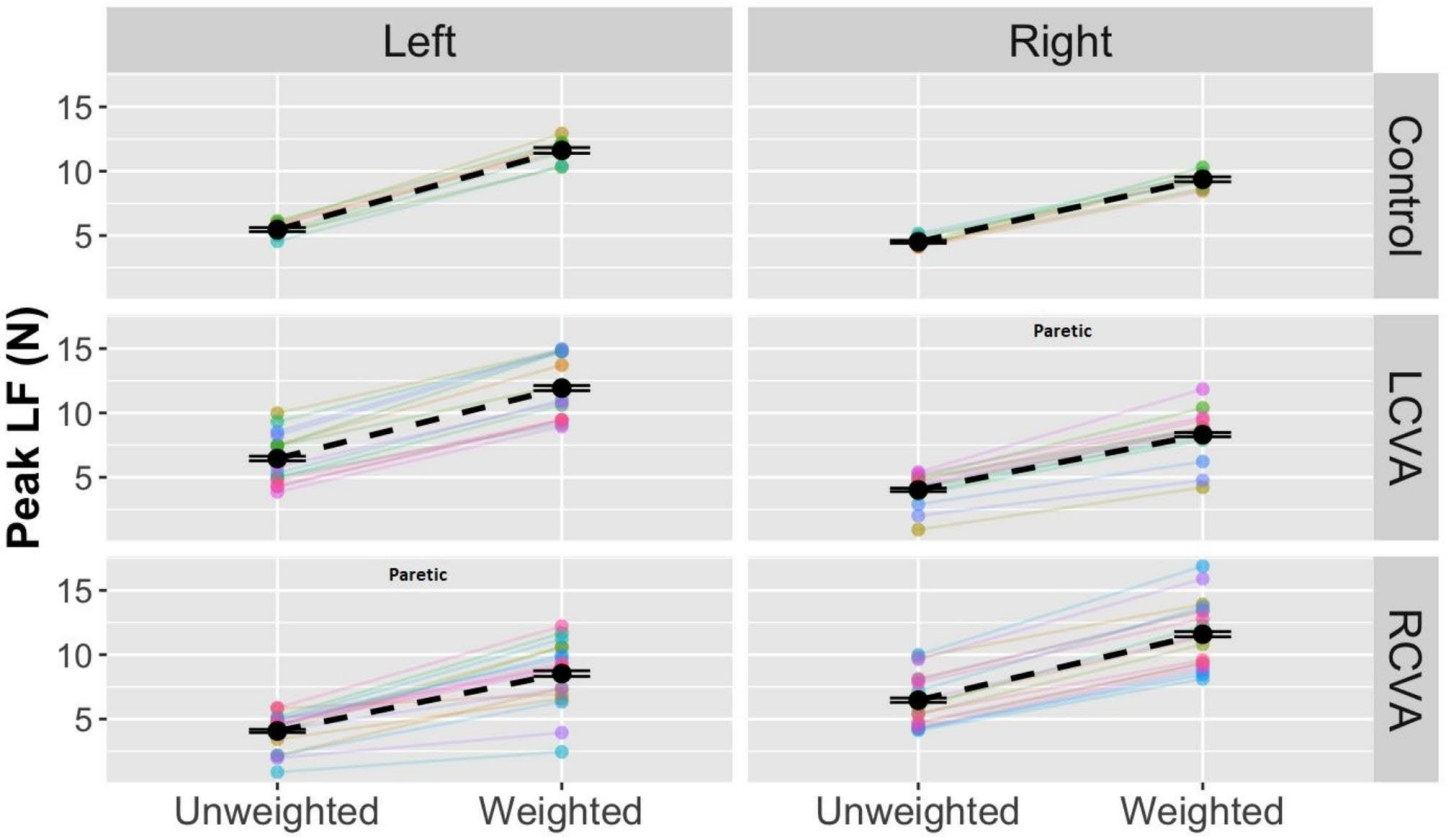
Peak LF plotted across Weight Condition by Group (Control, LCVA, RCVA) and Hand. Paretic hands are denoted for the LCVA and RCVA groups. Black points and dashed lines show group data with standard error. Colored lines and points show individual participant data

**Peak LF**. The analysis for peak LF revealed a significant three-way interaction between Group, Weight Condition, and Hand, χ^2^(2) = 12.33, *p* < .001. As shown in Fig. 4, control participants showed the largest change in peak LF across weight conditions as compared to either stroke group, and the magnitude of scaling was greater for the nondominant arm as compared to the dominant arm in controls and in the nonparetic as compared to the paretic arm for the LCVA and RCVA groups. Accordingly, there was also a significant interaction between Group and Hand, χ^2^(2) = 387.58, *p* < .001, a main effect of Hand, χ^2^(1) = 6.18, *p* < .05, and a main effect of Weight Condition, χ^2^(1) = 658.03, *p* < .001.

### Measures of within-hand coordination of grip and load force

We predicted that participants with stroke would show deficient coordination as compared to controls with increasing task demand, and that these effects may be more pronounced in the RCVA group based on prior work showing deficiencies in coordination with disruptions to right hemisphere structures. To examine differences in the coordination of force within either hand, we analyzed GF-LF correlation coefficients using a model with terms for Group, Weight Condition, and Hand.

**Fig. 5:**
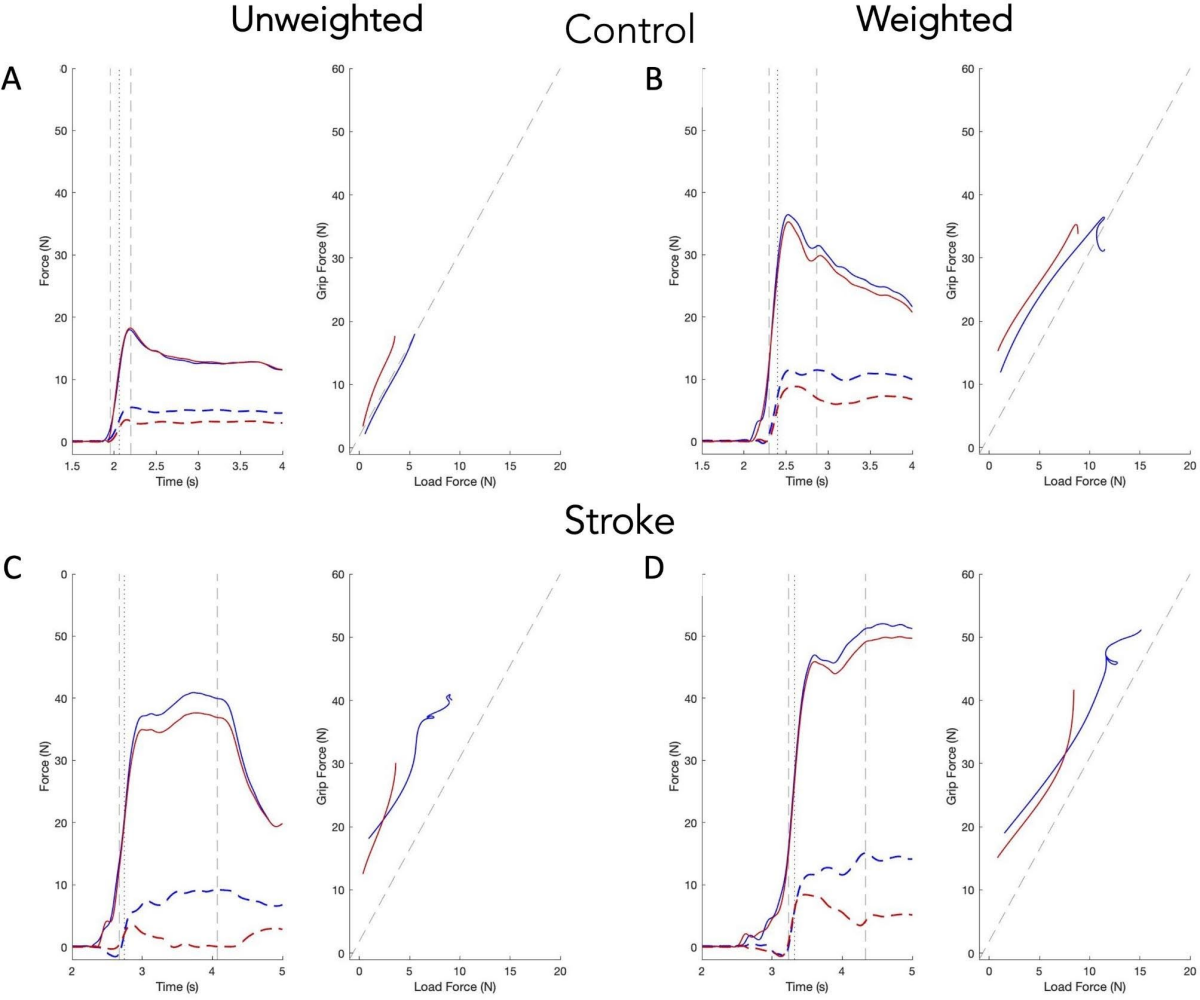
Each pair of plots shows GF (solid) and LF (dashed) as a function of time and GF plotted against LF for a single trial for a representative control participant (top) and a participant with stroke (bottom) in the unweighted (left column) and weighted (right column) condition. Blue lines show the left hand. Red lines show the right hand. Note that the participant with stroke has left CVA, thus the right hand (red) is paretic. LF onset and max are denoted using vertical dashed lines. Box liftoff is shown in the vertical dotted line

**GF-LF correlation**. Figure 5 shows GF and LF as a function of time (left panels) and GF against LF (right panels) for a representative control participant (Fig. 5A and B) and a participant with stroke (LCVA; Fig. 5C and D). For control participants, increases between GF and LF were approximately linear. Participants with stroke, however, showed significant variation in the coordination of these forces. As demonstrated in Fig. 5C and D, some individuals with stroke show marked impairments in LF production particularly with the paretic arm (i.e., the right hand in Fig. 5C and D).

**Fig. 6:**
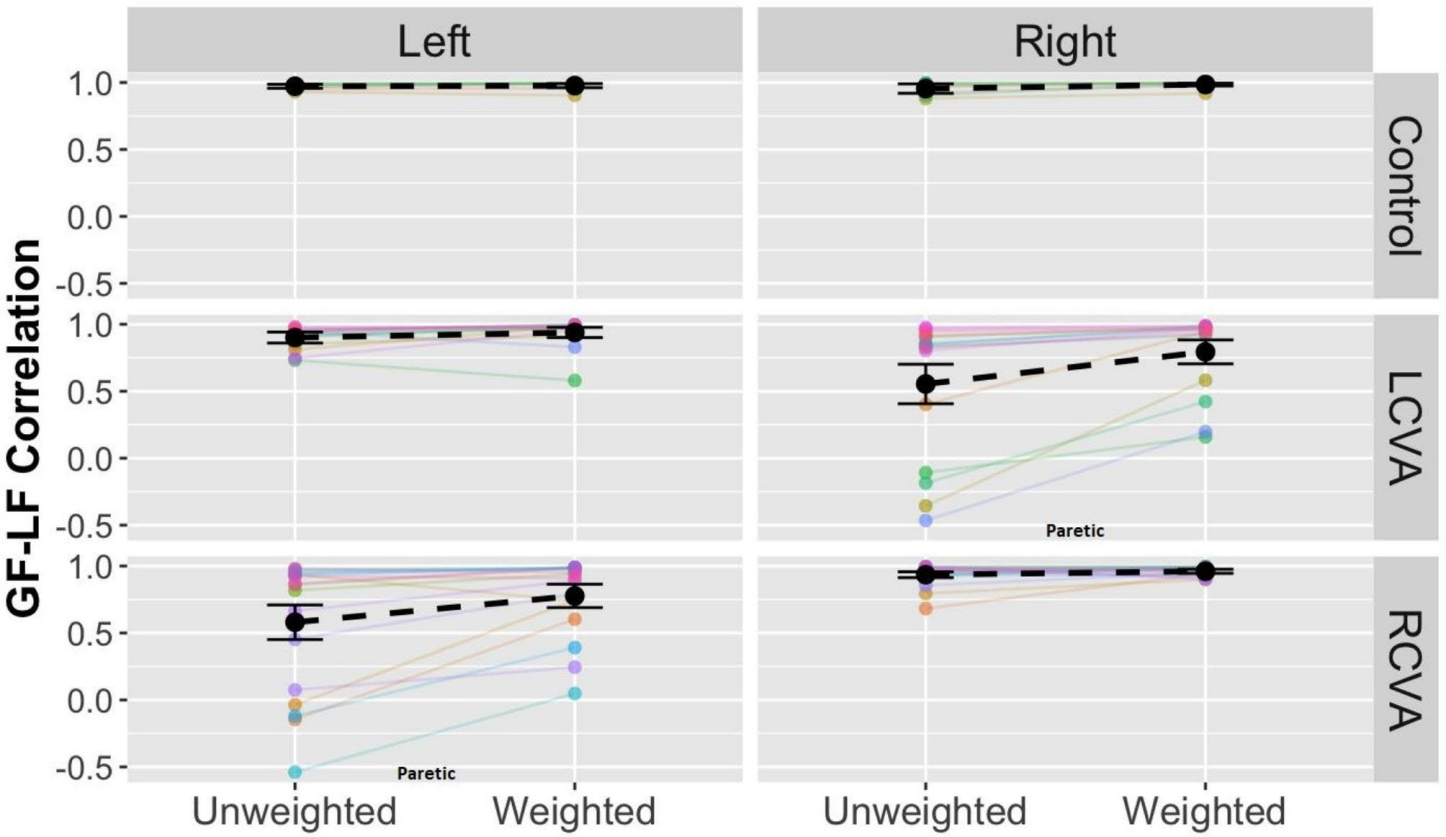
Grip-load correlation coefficients across weight condition by Group and Hand

The analysis of grip-load force correlation coefficients revealed significant interactions between Group and Condition, *χ*^*2*^(2) = 6.15, *p* < .05, and Group and Hand, *χ*^*2*^(2) = 138.28, *p* < .001, with no three-way interaction between Group, Weight Condition, and Hand or Hand by Weight Condition interaction (p > .05). These interactive effects are shown in Fig. 6. For the Group by Weight Condition interaction, both the LCVA and RCVA groups showed larger differences in grip-load coordination across weight conditions as compared to controls. The interaction between Group and Hand was driven by the higher grip-load correlation coefficients for the nonparetic left hand as compared to the paretic right hand in the LCVA group and the nonparetic right hand as compared to the paretic left hand in the RCVA group, while the two hands were similar for controls. There were also significant main effects of Hand, *χ*^*2*^(1) = 5.73, *p* < .05, Group, *χ*^*2*^(2) = 9.17, *p* < .05, and Weight Condition, *χ*^*2*^(1) = 21.10, *p* < .001.

In secondary analyses, we examined predictors of individual variability in GF-LF coordination among participants with stroke using backward stepwise regression with the absolute difference in GF-LF correlation between the two arms (nonparetic-paretic) as the outcome measure and hemisphere of lesion, paretic arm ARAT score, monofilament test score, age, and sex as predictors. The best-fitting model, *R*^*2*^ *=* 0.62, *F*(5, 20) = 9.1, *p <* .001, retained participants’ paretic arm ARAT score (*ß* = − 0.99, *p* < .001), sex (*ß* = − 0.35, *p* = .02), paretic side monofilament score (*ß* = − 0.23, *p* = .10), age (*ß* = 0.21, *p* = .14), and proprioception difference (*ß* = 0.19, *p* = .17).

***Bimanual coordination of force: Cross-correlation and time lag***. To examine the bimanual coordination of force, we analyzed GFR and LFR cross correlation and time lags in separate models with terms for Group, Weight Condition, and their interaction.

**Fig. 7:**
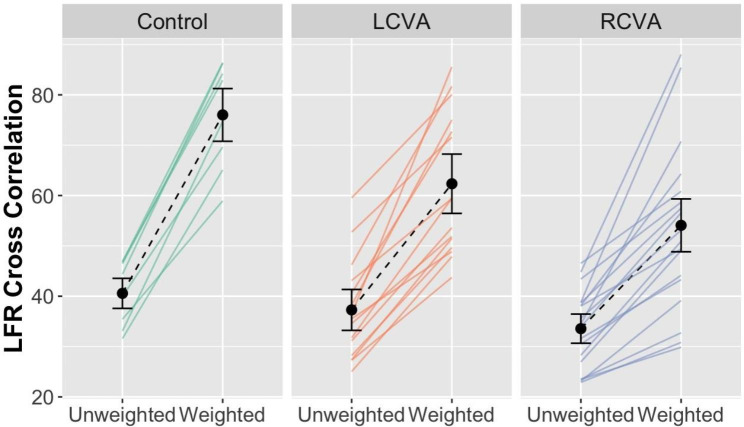
LFR cross-correlation coefficients across Weight Condition by Group

The analysis of between-hand cross correlations of LFR yielded a significant Group by Weight Condition interaction, χ^2^(2) = 17.17, *p* < .001 (Fig. 7). The RCVA group showed the largest difference in cross-correlation values across weight conditions (Unweighted: *r* = .71 *±* .45; Weighted: *r =* .84 *±* .24), followed by the LCVA group (Unweighted: *r* = .79 *±* .34; Weighted: *r =* .87 *±* .15) and then controls (Unweighted: *r* = .95 *±* .07; Weighted: *r =* .97 *±* .03). There was also a significant main effect of Weight Condition, χ^2^(1) = 76.35, *p* < .001. No significant effects emerged for the analyses involving LFR time lag, GFR cross-correlation coefficients, or GFR time lag values.

To examine predictors of bimanual coordination among participants with stroke, we analyzed mean LFR cross-correlation coefficient values in a backward stepwise regression with the same predictors as our previous analyses. The best fitting model, *R*^*2*^ = 0.62, *F*(3, 22) = 14.83, *p* < .001, included participants’ paretic arm ARAT score (*ß* = 0.83, *p* < .001), sex (*ß* =0.39, *p* = .006), and hemisphere of lesion (*ß* = 0.20, *p* = .09).

### Box tilt and hand position

The analyses of box tilt and hand position tested whether the between-group differences in measures of anticipation, execution, or coordination might be attributed to differences in the placement of the hands or how the box was lifted between groups.

**Tilt at Lift**. A one-sample t-test revealed that tilt at lift values differed from zero (no tilt), indicating significant box tilt at the time of lift, *t* [34] = -3.62, p < .001. We examined potential effects of Group and Weight Condition using linear mixed effects modeling. The analysis yielded a significant effect of Weight Condition, χ^2^(1) = 5.30, *p* = .02. Tilt at lift values were greater for the weighted box condition (-0.22 ± 0.76 cm) than the unweighted box condition (-0.09 ± 0.25 cm). Numerically, mean tilt at lift values suggested minimal leftward tilt for the control (-0.07 ± 0.44 cm), LCVA (-0.35 ± 0.87 cm) and RCVA (-0.10 ± 0.46 cm) groups. Importantly, there were no effects of Group (*p =* .26) or Group by Weight Condition interaction (*p* = .92).

**Tilt at Max Height**. Tilt at max height values did not differ significantly from zero, suggesting minimal tilt (*p =* .16). LME analysis revealed a significant effect of Weight Condition, χ^2^(1) = 6.29, *p* = .01, with no effect of Group (*p =* .62) or Group by Weight Condition interaction (*p* = .67). Mean tilt was greater for the weighted (0.51 ± 2.96 cm) as compared to the unweighted (0.07 ± 0.51 cm) box condition. In terms of the direction of tilt by group, controls showed positive mean tilt (0.14 ± 3.25 cm), suggesting rightward tilt toward the dominant arm, while participants with stroke showed tilt toward the non-paretic arm. Participants with LCVA showed negative (leftward) tilt (-0.06 ± 1.67 cm), while participants with RCVA showed positive (rightward) tilt (0.77 ± 2.42 cm). As demonstrated by the large standard deviations, however, there was considerable individual variation in box the direction and magnitude of tilt.

**Hand Position Differences at Box Lift**. To examine whether the tilt at box lift emerged from differences in hand placement on the box, we examined differences in left- and right-hand Z-position at box lift. Differences in left and right-hand Z-positions did not differ significantly from zero (*p* = .71). LME yielded no significant effects for Group (*p* = .64), Weight Condition (*p* = .93), or Group by Weight Condition interaction (*p* = .86). Mean hand difference values, while highly variable, suggested a tendency to grasp lower with the left than the right for the control (-0.36 ± 0.99 cm) and LCVA (-0.64 ± 1.32 cm) groups, while the mean for the RCVA group suggested lower right-hand position relative to the left (0.31 ± 2.37 cm).

## Discussion

Naturalistic actions such as picking up a box with both hands require the two hands to interact in cooperative coordination, planning and synchronously applying forces to grip and lift the box while adapting to changing object properties to ensure a smooth pickup. The present study was designed to investigate the anticipatory planning, execution, and coordination of forces between the hands as individuals with LCVA, RCVA and age-matched controls picked up a box under varying weight conditions. Our results revealed deficits in anticipatory planning, as indicated by group differences in the scaling of peak GFR and LFR across box weight conditions, as well as impaired bimanual coordination, as indicated by lower LFR cross-correlations between the two arms for participants in the RCVA group compared to controls and LCVA. Both stroke groups showed reduced within-hand grip-load force coordination for the paretic hand. Below we discuss differences across the two hands, predictors of individual variation among participants with stroke, and the relation between the present results and prior investigations of bimanual coordination after stroke.

### Individuals with RCVA demonstrate impaired anticipatory scaling of force during the bimanual cooperative task

Scaling of peak GFR and LFR occurs prior to object lift, the time at which feedback about the weight becomes more robust, and hence is thought to be dependent on anticipatory control and informed by sensorimotor memory developed over 1–2 consecutive lifts [20]. As hypothesized, and similar to previous studies in unimanual grasp-lift tasks, neurotypical individuals scaled their GFR and LFR to box weight; with added weight they increased their GFR and LFR during a bimanual pickup task. Based on hemispheric specialization for unimanual actions and findings of Schaffer et al. [27] in individuals with stroke, we hypothesized that the LCVA group will show impaired anticipatory planning compared to controls and RCVA group. Contrary to our hypothesis, the RCVA group showed significantly poor scaling of grip and load force rates across object weight conditions compared to controls than the LCVA group. Kang and Cauraugh [9] reported that individuals with RCVA showed greater deficits in force accuracy and variability than those with LCVA during a bilateral isometric force matching task. Our findings extend those of Kang and Cauraugh to more naturalistic tasks and suggest that right hemisphere may preferentially contribute to planning of grip force rates in bimanual actions. Our findings, however, contrast with those of Schaffer and colleagues [27], who demonstrated that individuals with LCVA show pronounced deficits in early phases of bimanual coordination as they navigate a virtual cursor in a planar bimanual reaching task. Besides differences in the environment (virtual vs. naturalistic), these differences can be attributed to the nature of the bimanual task between the two studies. In the kinematic task employed by Schaffer and colleagues [27], the key differences between LCVA, RCVA and controls were observed in arm movements along the redundant but not non-redundant axis of the task. In the more naturalistic task employed here, redundant movements were substantially constrained by box characteristics and instructions specifying hand placement and action goals. Such naturalistic bimanual actions that rely on specific positional control for planning may preferentially engage both hemispheres [35]. Previous work suggests that bimanual control is distinct for kinematic and kinetic actions as well as influenced by task goals [10], and is likely to be task-dependent.

There were no differences in sensorimotor impairments (scores on ARAT, UEFM, Semmes-Weinstein monofilament test or grip strength) between the RCVA and LCVA groups. Thus, these differences in LCVA and RCVA may arise from the specialized role of the right hemisphere in cooperative bimanual coordination. While bilateral neural networks are engaged in bimanual coordination, mounting evidence from imaging and TMS-perturbation studies point to the role of right PMd (premotor cortex) and right STG connectivity in movement planning and execution of cooperative bimanual actions [26, 36, 37]. For example, in healthy individuals, rTMS over right PMd during both planning and execution induced deterioration of movement stability of a complex cooperative bimanual task [37]. Similarly, interhemispheric connectivity of right M1 with contralateral left PMd during movement preparation has been shown to be related to bimanual performance [38]. Further, preferential and causal engagement of right STG has been reported for execution of cooperative bimanual actions [26]. Our results showing poorer scaling of LFR and GFR, as well as poor LFR coordination in individuals with RCVA provides further support to the notion that the right hemisphere may play a specialized role in planning and execution of bimanual cooperative actions. Further research investigating the neuroanatomic underpinnings using lesion symptom mapping and tractography may help elucidate the differential role of each hemisphere in bimanual actions, as well as provide additional insight into the task characteristics that preferentially engage these distinct modes of bimanual control.

Previous work in unimanual precision grasping has demonstrated anticipatory planning impairments in the paretic arm [21]. These deficits may arise from impairments in sensory perception, the ability to utilize sensory feedback to successfully form or update object representations [20, 34], judgements about the sense of effort [39], or higher-order motor planning to utilize sensorimotor memory to modify grasp behaviors [20]. In our analyses across both stroke groups, tactile sensory impairment, as measured with the Semmes-Weinstein monofilament test score, was retained in the stepwise regression for the scaling of GFR to object weight, suggesting some improvement of model fit, but was not a significant predictor of deficient scaling. In contrast, the scaling of GFR was significantly associated with the paretic arm ARAT score, suggesting that the scaling of force rate was associated with motor performance deficits of the paretic arm. Such motor impairment may lead to abnormal grasping postures with the paretic hand that may hinder the ability to effectively utilize sensory feedback to inform the sense of effort and sensorimotor memory. Further, neuromuscular deficiencies observed in individuals with stroke such as the delayed moto-neuron recruitment observed after corticospinal tract damage may also result in slowed force rates [40, 41]. These deficits in the paretic arm, combined with known higher-order planning impairments in both the paretic and non-paretic arm may contribute to impaired scaling of GFR and LFR in the bimanual box pick up task.

### Impaired paretic arm production and coordination of load force affects execution of cooperative bimanual tasks after stroke

Differences between the two hands were evident in both the control group and the stroke groups. The control group showed steeper increases in peak LF and LFR across weight conditions for the nondominant left than the dominant right hand. Moreover, mean tilt values indicated rightward tilt at max height, suggesting that the dominant arm may have been used for stability. This was a surprising finding, particularly in light of previous studies supporting the specialized role of the dominant right hand in dynamic control as compared to a greater stabilization role of the nondominant left hand during unimanual as well as asymmetric bimanual actions (42). Prior research has shown that during bimanual manipulation, healthy controls show covariation of load forces and hand placement to ensure successful performance (19). Thus, one possibility is that the asymmetry in LF and LFR could have been driven by asymmetric hand placement, with the left hand at a lower level than the right hand while picking up the box. The kinematic data, however, revealed no significant differences in the placement of the left and right hands, suggesting that participants followed the specified instructions and visual cues for hand placement during task performance. Additionally, the weight added to the box was secured within a narrow channel, and thus it is unlikely that these effects were due to shifts in the weight’s position inside the box. However, despite clear instructions, we observed that the box was tilted at box lift. This box tilt may have been mediated by asymmetry in the LF and LFR seen in the control group.

In terms of differences between the two hands, participants with LCVA and RCVA showed greater impairments in the scaling of LF and LFR for the paretic compared to the nonparetic arm, though effects of hand did not appear for GF or GFR. Additionally, there was significantly impaired within-hand GF-LF coupling for the paretic arm as compared to the nonparetic arm. Careful observation of the relation between GF and LF for individual trials (e.g., the participant with stroke in Fig. 5) suggests a particular deficit in the production and temporal evolution of load force, as compared to grip force, for the paretic arm compared to the nonparetic arm. Similarly, spatial coordination between the two arms for LFR was significantly impaired in the stroke group, suggesting deficits in the covariation of force across the paretic and nonparetic arms. Importantly, paretic arm decrements in GF-LF coupling as well as cross-correlation coefficients for LFR were predicted by the level of motor (i.e., ARAT) and sensory (Semmes-Weinstein test) impairments. Motor deficits such as longer times to peak force as well as lower peak forces, evident in the paretic arm, may contribute to impaired load force evolution and LFR coordination. Further, proximal arm kinematics in neurotypical individuals is known to affect GF-LF coupling in unimanual grasping [43]. Poor scores on UEFM are often associated with abnormal kinematics of shoulder and elbow during reaching, that may subsequently impair GF-LF coupling for the paretic arm [44]. The finding that sensory impairment was also associated with poor GF-LF coupling is in alignment with prior findings from our and other laboratories and highlight the role of sensory information in the execution of cooperative coordination of a bimanual task [18, 45]. Grip force rate scaling is known to be related to a central sense of effort gathered from sensory information from the previous trial [20] that, in a bimanual task such as the one tested here, may arise from both the paretic and nonparetic hand. Thus, stroke survivors may be able to use the information from the nonparetic arm to inform grip force scaling of both hands during subsequent trials, consistent with unimanual work showing paretic arm improvements in force production following lifts with the nonparetic arm [21]. On the other hand, the stroke groups produced greater peak LFR and higher LF values with the nonparetic arm. This suggests that the paretic arm was used to stabilize the box, while the nonparetic arm produced the tangential forces needed to counter gravity for box lift. Collectively, our data suggests that individuals with stroke demonstrate deficits in execution of cooperative task of picking a box, and these deficits, in part, can be attributed to poor control of LF and LFR secondary to sensory and motor impairments of the paretic arm. Future research that systematically manipulates the center of mass of the box while measuring grip and load force profiles may reveal how the two arms compensate for one another during cooperative bimanual tasks following stroke.

### Interlimb coordination is impaired after stroke and provide unique insight into arm use strategies under bimanual actions

Finally, we also observed that, in addition to differences between the control and stroke groups, the cooperative coordination of LFR was different across the two weight conditions. Cross-correlation coefficients for LFR were significantly higher for weighted trials as compared to unweighted trials for controls and participants with stroke, suggesting that the coordinated production of force between the two arms was improved when task demands were increased through added weight. Similarly, GF-LF coordination improved for the weighted as compared to unweighted box condition for both groups. This suggests that patients likely have the capacity to coordinate LFRs between the hands, however, that capacity may be masked until task demands require them to do so (46). This finding contrasts with prior work from Kang and Cauraugh (7) reporting greater force variability in bimanual isometric force production at 50% but not 25% or 10% maximum voluntary contraction (MVC). Thus, it is possible that the weight of the box alone was insufficient to elicit 50% MVC. Alternatively, object properties and task goals may constrain the two arms differently than the isometric force production task. In an unweighted box, the paretic arm may contribute more to supporting the object while the nonparetic arm applies load forces to pick up the box. As the weight increases, the paretic hand can contribute to the production of load force in coordination with the nonparetic arm. This latent capacity to coordinate LFR between two arms in the weighted condition, but not in the unweighted condition, is akin to the phenomenon of learned non-use or reduced use. Learned nonuse is conceptualized as the disparity between the arm use and actual capacity of the paretic arm in stroke survivors (47). Though non-use (or reduced use) has primarily been studied in unimanual actions, our data suggest that gradations of ‘arm nonuse’ may arise even when the paretic arm is actively engaged during a cooperative bimanual task. However, the finding that LFR cross-correlations related to hemisphere of lesion and paretic arm ARAT scores suggests that the extent of this latent capacity may vary depending on the location of stroke and paretic arm function.

## Limitations

While we observed planning deficits in bimanual actions, without concomitant testing of unimanual actions, we cannot ascertain that these planning deficits are specific to bimanual actions and not an overarching planning deficit. Additionally, there were several parameters that were relatively unconstrained during the bimanual box-pickup task that may have affected results, including hand placement, tilt, and lift height. The control sample was relatively small (*N* = 8); thus, it is possible that results such as the hand differences in LF and LFR may have emerged from individual variation unique to this sample. There is no generally accepted method for estimating power achieved using linear mixed effects analyses, thus the present effects, and particularly three-way interactions, should be interpreted with caution [48]. However, this dataset can be utilized for more precise sample size estimations in future studies. Finally, stepwise regression suffers several limitations, including potential distortions in model fit related to covariation among predictors, and thus should be treated as exploratory.

## Clinical implications and conclusion

In summary, in the bimanual cooperative, common goal box pick-up task, individuals with chronic stroke, particularly those following RCVA differed from controls in the anticipatory scaling of force rate, as well as the coordinated production of force within and across the two arms. During execution, individuals with stroke showed specific impairments in the paretic arm for LF and LFR scaling, coordination between LF and GF, and LFR coordination between the two arms. These findings suggest that interventions targeting scaling of LF and coordination of interlimb LFR may be needed to improve bimanual performance and coordination. Impairments in planning were also evident in the scaling of GFR, suggesting the need for future work investigating whether naturalistic task practice under varied manipulations of object weight and size may improve GFR scaling during bimanual action. Future research is needed to elucidate the neural underpinnings of impairments in the anticipatory scaling of force after stroke, including the hemispheric differences that emerged in the bimanual box pick-up task. Finally, our results suggest that subtle gradations of arm non-use may emerge even when both arms are engaged and actively cooperating toward a common goal. Thus, rehabilitative strategies aimed to improve paretic arm participation should be careful to prevent such non-use from emerging, perhaps through adjustments to task demands.

### Electronic supplementary material

Below is the link to the electronic supplementary material.

Supplementary Material 1

## Data Availability

Data and supplementary materials for the current study are available from the corresponding author on reasonable request.

## List of Abbreviations

ARAT: Action Research Arm Test
CAT: Comprehensive Aphasia Battery
CVA: Cerebrovascular accident
GF: Grip force
GFR: Grip force rate
LF: Load force
LFR: Load force rate
LME: Linear mixed effects modeling
M1: Primary motor cortex
MVC: Maximum voluntary contraction
SMA: Supplementary motor area
SMMSE: Standardized Mini Mental State Examination
STG: Superior temporal gyrus
UEFM: Upper Extremity Fugl-Meyer
WAB: Western Aphasia Battery
