# Supplementary material for: Post-stroke deficits in the anticipatory control and bimanual coordination during naturalistic cooperative bimanual action
Source: J Neuroeng Rehabil. 2023 Nov 10;20:153. doi: 10.1186/s12984-023-01257-x (PMC10638820; doi:10.1186/s12984-023-01257-x)
Supplement: Supplementary file 1 — Supplementary Material 1 [file 12984_2023_1257_MOESM1_ESM.pdf]

Supplementary Data

Post-stroke deficits in the anticipatory control and bimanual coordination during naturalistic cooperative bimanual action

Table 1. Peak GFR: Group by Weight Condition means and SDs (N/s)

	Control	LCVA	RCVA
Condition	*M (SD)*	*M (SD)*	*M (SD)*
Unweighted	132.7 (37.8)	117.5 (78.2)	96.2 (45.9)
Weighted	212.4 (88.8)	178.2 (106.1)	131.6 (59.0)

Table 2. Peak LFR: Means and SDs (N/s)

	Control			LCVA			RCVA		
	Left	Right	Combined	Left (Nonparetic)	Right (Paretic)	Combined	Left (Paretic)	Right (Nonparetic)	Combined
Condition	*M (SD)*	*M (SD)*	*M (SD)*	*M (SD)*	*M (SD)*	*M (SD)*	*M (SD)*	*M (SD)*	*M (SD)*
Unweighted	44.0 (15.2)	40.9 (8.0)	42.5 (12.2)	41.1 (16.8)	33.5 (13.7)	37.3 (15.7)	31.1 (13.7)	36.0 (10.2)	33.6 (12.3)
Weighted	83.1 (.19.2)	71.9 (14.8)	77.5 (18.0)	66.0 (22.3)	56.2 (22.4)	61.1 (22.8)	49.3 (22.2)	56.2 (21.4)	52.7 (22.0)
Combined	72.0 (25.3)	63.1 (19.3)		58.7 (23.7)	49.6 (22.7)		44.0 (21.7)	50.3 (20.9)	

Table 3. Peak LF: Means and SDs (N)

	Control		LCVA		RCVA	
	Left	Right	Left (Nonparetic)	Right (Paretic)	Left (Paretic)	Right (Nonparetic)
Condition	*M (SD)*	*M (SD)*	*M (SD)*	*M (SD)*	*M (SD)*	*M (SD)*
Unweighted	5.4 (.7)	4.5 (.5)	6.5 (2.1)	4.0 (1.3)	4.1 (1.4)	6.4 (2.1)
Weighted	11.6 (1.0)	9.4 (.8)	11.9 (2.5)	8.3 (2.0)	8.7 (2.6)	11.5 (2.7)

Table 4. GF-LF mean correlation and SDs

	Control			LCVA			RCVA		
	Left	Right	Combined	Left (Nonparetic)	Right (Paretic)	Combined	Left (Paretic)	Right (Nonparetic)	Combined
Condition	*M (SD)*	*M (SD)*	*M (SD)*	*M (SD)*	*M (SD)*	*M (SD)*	*M (SD)*	*M (SD)*	*M (SD)*
Unweighted	.97 (.04)	.95 (.09)	.96 (.08)	.90 (.16)	.94 (.57)	.73 (.45)	.58 (.55)	.94 (.09)	.76 (.43)
Weighted	.98 (.04)	.98 (.03)	.98 (.03)	.94 (.13)	.96 (.35)	.87 (.28)	.79 (.35)	.96 (.06)	.87 (27)
Combined	.98 (.04)	.98 (.06)		.93 (.14)	.72 (45)		.72 (.44)	.95 (.08)	
